# Balanced cytokine upregulation by diluted ethanolic extract of *Bryonia alba* in Delta SARS-CoV-2 Spike protein RBD-induced pathogenesis in *Gallus gallus* embryo

**DOI:** 10.1186/s42269-022-00856-3

**Published:** 2022-06-13

**Authors:** Pritam Goswami, Debasmita Chatterjee, Sayak Ghosh, Krishnendu Paira, Satadal Das

**Affiliations:** 1 West Bengal University of Health Sciences, Kolkata, India; 2 Genetic Research Laboratory, Heritage Institute of Technology, Kolkata, 700 107 India

**Keywords:** SARS-CoV-2, *Bryonia alba*, Chick embryo, Immunomodulatory action

## Abstract

**Background:**

*Bryonia alba* extract is a well-known drug which is being utilized as phytomedicines and homoeopathic preparations since more than two centuries. This medicine is frequently used in clinical practice for flu-like conditions, respiratory tract infections, and gastrointestinal diseases, as evidenced by old literature and historical records. The plant contains Bryonicin, Bryonolic acid, Bryodin, Cucurbitacin, etc. The alkaloids in *Bryonia alba* have been discovered to be a powerful heme-oxygenase-1 inhibitor, which could help reduce oxidative stress during SARS-CoV-2 pathogenesis. During three waves of SARS-CoV-2, extracts of *Bryonia alba* were used; however, the actual scientific explanation for its mechanism of action is still unknown. In this experiment, we studied cytokine changes by diluted *Bryonia alba* extract in Delta SARS-CoV-2 spike protein RBD-induced pathogenesis, in fertilized chick (*Gallus gallus domesticus*) embryos.

**Results:**

The recombinant Delta SARS-CoV-2 spike RBD protein was inoculated in 14-day-old chick (*Gallus gallus domesticus*) embryos along with control, pre-, and post-treatment sets with diluted Bryonia extract. After 48 h, allantoic fluids were collected and stored at – 20 °C for study of different cytokines. Histological changes of the liver were also studied in each animal. Diluted Bryonia extract upregulated IFN-α and IL-10 markedly. In pre-treatment set, IFN-α, IL-8, IL-10, and IL-1β were markedly decreased, while in the post-treatment set IL-6, IL-10, IL-8, and TGFβ1 were significantly decreased, with a tendency of more anti-inflammatory surge than pro-inflammatory cytokines.

**Conclusions:**

This experiment indicated an immunomodulatory role of diluted ethanolic extract of Bryonia particularly in the post-treatment set, decreasing pro-inflammatory cytokines with beneficial effect.

## Background

Viruses are symbionts that exhibit parasitism, commensalism, and mutualism as a result of which they not only invade our tissues, but also leave a footprint in our DNA. The apocalypse named SARS-CoV-2 has already claimed the lives of 4.9 million people across 188 countries (http://www.worldometers.info^2021^). Only 20% of SARS-CoV-2 infected patients are symptomatic, and only 5% progressed to severity. However, those that do are faced with a lack of definite targeted therapy, exacerbating an already difficult situation.

In addition to conventional medicine, CAM has also been used to treat patients with mild to moderate symptoms in different parts of the globe (Lam et al. 2021; Charan et al. 2020; Stub et al. 2020). However, like modern medicine, the drugs that have been used in CAM need thorough scientific exploration. This will help to determine their scope and limitations for better implementation. The cytokine profile of COVID-19 patients reveals a link between the severity of the disease and a hyper-inflammatory state. Interleukins(IL)-2, 6, and 10, granulocyte–macrophage colony-stimulating factor (GM-CSF), interferon-induced protein (IP)-10, monocyte chemoattractant protein (MCP)-1, macrophage inflammatory protein (MIP)-1A, and TNF-α are among the cytokines that are significantly enhanced in COVID-19 patients (Huang et al. 2020; Zhu et al. 2020). SARS-CoV-2 upregulates IL-6, which plays a significant role in severity and morbidity. It forms a complex with the membrane-bound IL-6 receptor (mIL-6R) or the soluble IL-6 receptor (sIL-6R), which ignites the inflammation (Panigrahy et al. 2020). Besides that elevated level of IL-6 is also responsible for the secretion of acute-phase reactants, like serum ferritin, C-reactive protein (CRP), complements, and pro-coagulant factors, which may worsen the prognosis (Bhaskar et al. 2020). Moreover, increased inflammatory cytokines are associated with reduced T-lymphocytes in peripheral circulation and slow down the process of virus clearance (Favalli et al. 2020). To combat this vicious cycle, several therapeutic attempts, including immunomodulatory drugs, IL-6 inhibitors, and plasma therapy, have been used, but without significant impact on the disease. This leads to host immunity as the principal barrier against the virus. Even though CAM was utilized on a limited cohort, the drug *Bryonia alba* was frequently prescribed to COVID-19 patients with favourable results, which initiated our quest for this experiment (To and Fok 2020; Jethani et al. 2021). In this experiment, we attempted to find out the effect of a diluted ethanolic extract of *Bryonia alba*, in Delta SARS-CoV-2 spike RBD protein-induced systemic inflammation in chick (*Gallus gallus domesticus*) embryo.

### Bryonia phytochemicals

**Fig. 1:**
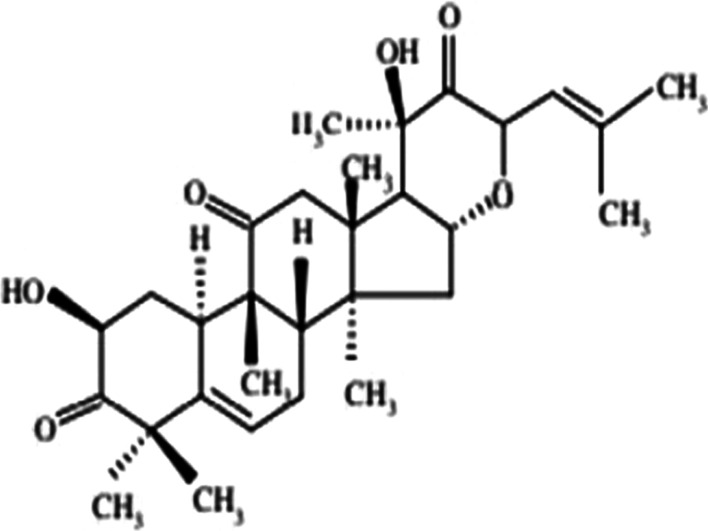
Molecular structures of some bio-active agents of *Bryonia alba* L. 22-deoxocucurbitosides A

**Fig. 2:**
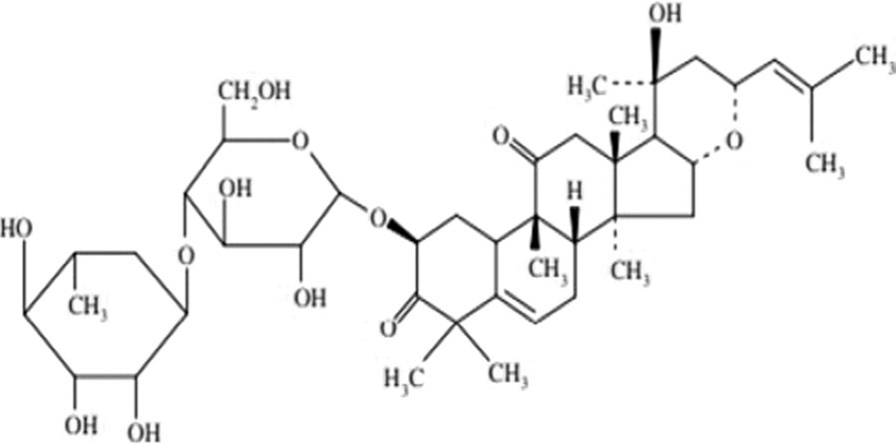
22-deoxocucurbitosides D

**Fig. 3:**
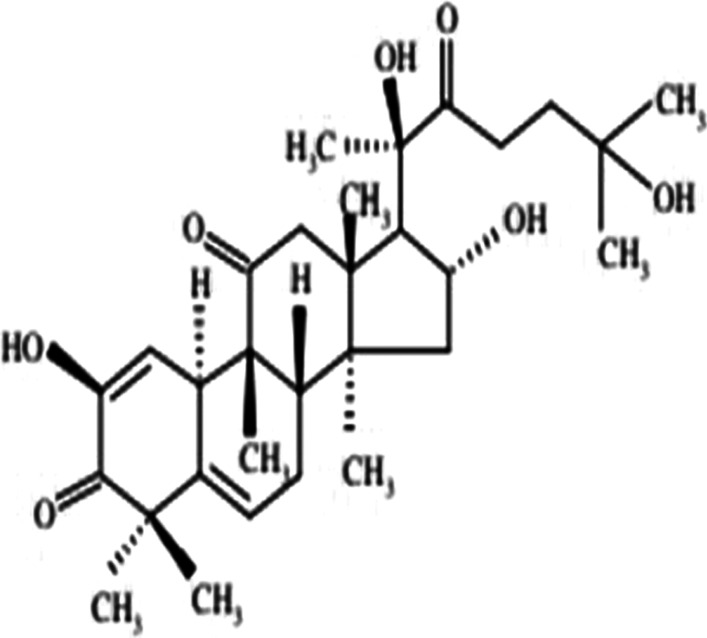
Cucurbitacin L

**Fig. 4:**
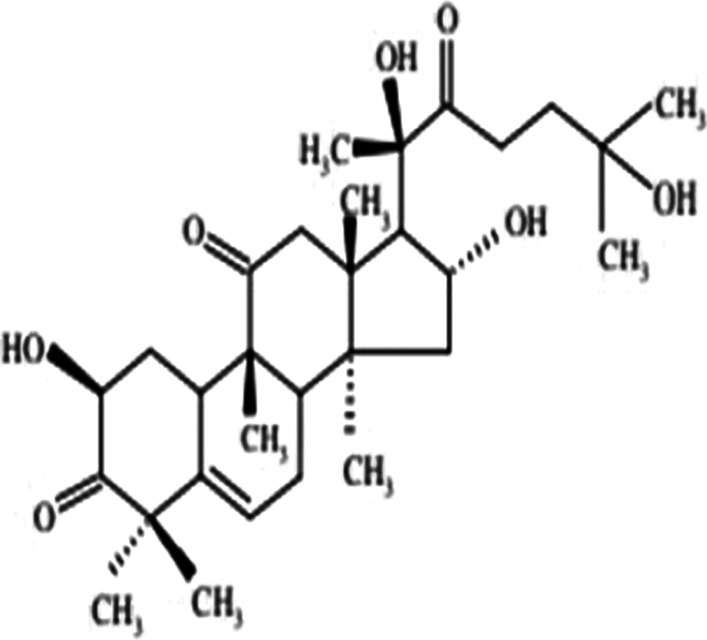
23,24-dihydrocucurbitacin D

**Fig. 5:**
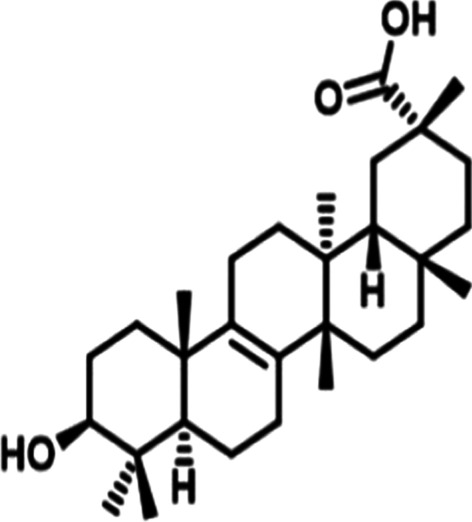
Bryonolic acid (BA) 3β-hydroxy-D:C-friedoolean-8en-29-oic acid

*Bryonia alba* (White Bryony) is a commonly used homoeopathic medicine that has been used in folklore and traditional medicine since last two millennia. Bryonia is one of the smallest genus of the family Cucurbitaceae, where *Bryonia alba* Linn and *Bryonia dioica* Jacq have been recorded in the literature for their use in traditional medicine. *Bryonia alba* L. is mostly distributed in Europe and West Asia. The juice of this perennial climber plant found beneath its root is known to cure oedema, convulsions, headaches. Ointment prepared from the juice relieved pneumonia as reported in the folklore literature. Apart from that, it also has been used in inflammation of the serous tissue, cough, peritonitis, jaundice, rheumatism, and in brain disorders (Kujawska and Svanberg 2019; Manvi and Garg 2011; Lee et al. 2013). Phytochemical studies suggest *Bryonia alba* L. contains alkaloid bryonicine; glycosides 22-deoxocucurbitosides A (Fig. 1); and B, 22-deoxocucurbitosides D (Fig. 2). It also contains triterpenoids named cucurbitacin L (Fig. 3), 23,24-dihydrocucurbitacin D (Fig. 4); flavonoids saponarin, lutonarin, isoorientin, vitexin, isovitexin, 5, 7, 4’-trihydroxy flavone 8-C-glucopyranoside. Cucurbitacin is a group of tetracyclic triterpenes found in the plant that holds various ethnopharmacological properties. Several in vitro and in vivo experiments have shown the anti-inflammatory, anti-cancer, cytoprotective, anti-diabetic activities (Ilhan et al. 2019; Gatbonton-Schwager et al. 2012; Wang et al. 2015). Cucurbitacin also has proven to have immunosuppressive effects as it interferes with the adaptive immunity by downregulating the NF-κB pathway (Wang et al. 2015; Xie et al. 2020). Interestingly, in a recent study Cucurbitacin has evolved as great therapeutic agent, where it showed its analogous property with the SARS-CoV-2. Cucurbitacin can inhibit the JAK/STAT 3 pathway which eventually stops the recruitment of T-lymphocyte, preventing the hyper-inflammatory state in COVID-19 (Kapoor et al. 2020; Wang et al. 2014). Bryonolic acid (BA) 3β-hydroxy-D:C-friedoolean-8en-29-oic acid (Fig. 5) is another bio-active compound found in the roots of *Bryonia alba* L. (Visansirikul and Lertphadungkit XXXX). Previous studies have revealed many biological effects of *Bryonia alba*. It also possesses the anti-allergic, anti-cancer, anti-oxidation, anti-inflammatory activities like the other flavonoids and triterpenoids. In this context, it appears necessary to study different aspects of its chemical and biological activities, especially as it is reported to have therapeutic uses (Visansirikul and Lertphadungkit XXXX; Rus et al. 2015). Not only is this species reported to have important homoeopathic uses, but it has also been reported to have many traditional uses. Thus, in traditional medicine it is used as antipyretic, anti-inflammatory, antibacterial, laxative-purgative, smooth muscle relaxant, cytotoxic, hepato-protective, and anti-diabetic agent (Rus et al. 2015), which prove its promising potential for the treatment of various diseases.

## Methods

### The recombinant antigen

Delta SARS-CoV-2 spike RBD (L452R, E484Q) protein, manufactured by ABclonal Lot: 9621050601, Cat. No. RPO2266, Code: WH192258, was procured for this experiment. The HEK 293 expression system was used to make this recombinant protein. The target protein was made up of the SARS-CoV-2 spike RBD sequence (Arg 319-Phe541) bound to a poly-histidine tag at the C-terminus (with mutations L452R, E484Q, Accession #YP-009724390.1). The mutations were identified in the SARS-CoV-2 variant (known as variant B.1.617), which emerged in the India. The lyophilized protein was 0.22 μm filtered solution in PBS, pH 7.4. It produced a band in SDS-PAGE at 35 kDa.

### The embryonated eggs

Thirteen-day-old embryonated *Gallus gallus domesticus* chicken eggs were procured from Government State Poultry Farm, Kolkata, India, which was free from any pathogen. The surface of the eggs was cleaned with distilled water followed by application of rectified spirit. All the eggs were candled and incubated at 38°C in 60–80% humidity. On 14th day, the eggs were arranged in six different groups. Except the normal control, in all groups 100μL of each material was injected through amniotic route. The eggs were candled next day of inoculation, rotated three times throughout the day, and incubated as mentioned above. Harvesting of all the eggs was done after 48 h (on 16th day) following exposure at 2–8 °C for 2 h. 5–10 mL of allantoic fluids was collected in sterile vials and stored at −80 °C for further analysis. Physical conditions of the embryo were also observed, and tissues from the liver were collected for biopsy and observed under light microscope after H&E staining.

### Medicines and vehicle alcohol

*Bryonia alba* 6C, which contains < 1 pg concentration of crude extract, was directly purchased from Dr. Willmar Schwabe India Pvt. Ltd., a GMP certified company. It follows standard Government guideline for the preparation of medicines.

### Estimation of expressions of the cytokines

Comparative gene expression studies were determined after real-time PCR (Bio-Rad CFX96, Singapore) with SYBR Green tagged primers, dNTPs, Taq polymerase, MgCl2, buffer, etc. The changes of expressions were calculated as fold increase or decrease from the value of the normal control and compared with house-keeping gene β-actin.

## Results (Table 1)

Delta SARS-CoV-2 spike protein RBD antigen, in concentration of 10 µg/mL, increases IFN-α, more than 1500 times. It also increases IFN-ß about 150 times, and TGF-ß about 74 times. Increase in IFN-ƴ, IL-6, IL-8, IL-10, and IL-1ß was insignificant.

**Table 1:** Increase in cytokines (fold increase in comparison with normal control) in different experimental sets after 48 h

SETS	IFN-α	IFN-ß	IFN-ƴ	IL-8	IL-10	IL-1 B	TGF B1	IL-6
Bryonia 6C	66,129	893	5	88	1666	101	1.41	118
Bryonia 6C to Antigen	464	56	36	0.4	4	0.4	0.01	21
Antigen to Bryonia 6C	2179	24	2	0.4	9	5	0.00	5.7
Antigen	1692	149	2	3	2	2	74	7
Alcohol (70%)	116	36	2	3	0	2.4	3	59

When after administration of Bryonia 6C, the chick embryo was challenged with antigen, only interferons are moderately upregulated along with IL-6. Although increase in interferons after 48 h is beneficial to the embryo, an increase in IL-6 may lead to initiation of upsurge of pro-inflammatory cytokines. This may be detrimental to the normal physiological activities of the embryo, and thus, the preventive role of Bryonia 6C is questionable. In the curative experiment after administration of antigen, and then challenging with Bryonia 6C, the upregulation of IL-6 was decreased about 4 times than in the preventive study. Along with that, there was marked increase in IFN-α (5 times more as compared to the preventive group), and there was minor increase in IFN-ß. Thus, the curative role of Bryonia 6C in immune-pathological changes induced by Delta SARS-Cov-2 spike RBD was significantly more. Thus, it is expected that the side effects would be much less.

### Histological changes

#### Architecture

There were no architectural changes of the liver lobules except few pseudo-lobular structures in the liver of direct antigen set embryos.

#### Hepatocytes

Few ballooning degenerations and Mallory–Denk-like bodies are present in hepatocytes, where antigen was directly administered, while many Mallory–Denk-like bodies are present in hepatocytes in pre-treatment set experiment.

#### Other changes

Lobular necroinflammation (acute hepatitis), apoptosis, massive bridging necrosis, interface hepatitis (Batts–Ludwig grade 3), portal mononuclear cell infiltration are present in experimental set with direct antigenic challenge. Mild necrosis, apoptosis, mild interface hepatitis (Batts–Ludwig grade 1), and few portal mononuclear cells are present in both pre- and post-treatment experimental sets.

#### Statistical analysis

There are significant correlations between IFNα and IL-1β, IL-6 and IFNα, IL-8 and IFNβ, TGFβ1 and IFNβ, TGFβ1 and IL-8, IL-6 and IL-8. These results signify normal relationship between them which was reflected in this experiment.

**Table:** 

Correlations							
	I	II	III	IV	V	VI	VII
II	0.282						
III	− 0.074	− 0.190					
IV	0.601	0.922	− 0.046				
V	0.457	− 0.167	− 0.155	− 0.014			
VI	0.837	0.614	− 0.200	0.817	− 0.012		
VII	− 0.053	0.924	− 0.138	0.732	− 0.189	0.267	
VIII	0.808	0.306	0.213	0.595	0.549	0.583	0.096

Pairwise Pearson correlations							
Sample 1	Sample 2		Correlation		95% CI for ρ		*P*-value
II	I		0.282		(− 0.470, 0.797)		0.462
III	I		− 0.074		(− 0.703, 0.621)		0.851
IV	I		0.601		(− 0.106, 0.904)		0.087
V	I		0.457		(− 0.297, 0.860)		0.216
VI	I		0.837		(0.391, 0.965)		0.005
VII	I		− 0.053		(− 0.693, 0.633)		0.892
VIII	I		0.808		(0.310, 0.958)		0.008
III	II		− 0.190		(− 0.759, 0.542)		0.624
IV	II		0.922		(0.667, 0.984)		0.000
V	II		− 0.167		(− 0.748, 0.559)		0.668
VI	II		0.614		(− 0.084, 0.908)		0.078
VII	II		0.924		(0.672, 0.984)		0.000
VIII	II		0.306		(− 0.450, 0.806)		0.424
IV	III		− 0.046		(− 0.689, 0.638)		0.906
V	III		− 0.155		(− 0.743, 0.568)		0.691
VI	III		− 0.200		(− 0.763, 0.535)		0.607
VII	III		− 0.138		(− 0.735, 0.579)		0.724
VIII	III		0.213		(− 0.526, 0.768)		0.582
V	IV		− 0.014		(− 0.672, 0.656)		0.972
VI	IV		0.817		(0.335, 0.960)		0.007
VII	IV		0.732		(0.133, 0.939)		0.025
VIII	IV		0.595		(− 0.114, 0.903)		0.091
VI	V		− 0.012		(− 0.671, 0.657)		0.975
VII	V		− 0.189		(− 0.758, 0.543)		0.626
VIII	V		0.549		(− 0.181, 0.889)		0.126
VII	VI		0.267		(− 0.482, 0.791)		0.487
VIII	VI		0.583		(− 0.133, 0.899)		0.100
VIII	VII		0.096		(− 0.607, 0.715)		0.806

Where the numbers I–VIII denote the following: I—Interferon alpha (IFN α), II—Interferon beta (IFN β), III—Interferon gamma (IFN γ), IV—Interleukin 8 (IL -8), V—Interleukin 10 (IL-10), VI—Interleukin 1 beta (IL-1β), VII—Transforming growth factor beta 1 (TGF-β1), VIII—Interleukin 6 (IL -6).

## Discussion

The term “Cytokine Storm” was first used in medical literature in a case of acute graft-versus-host disease following allogeneic hematopoietic stem-cell transplantation 28 years ago. But clinically, it was limited to influenza-like syndromes that develop secondary to sepsis or immunosuppressive therapies by Coley's toxins (Ferrara et al. 1993; Coley 1893). The present epidemic has also been added to the list, as the cytokine profile of SARS-CoV-2 patients reveals a hyper-inflammatory state. In humans, the pathogenesis of SARS-CoV-2 can be split into two parts, the first of which is the “immune system medicated protective phase”, and the second of which is the “inflammation mediated damaging phase” (Shi et al. 2020). Till now to control the viral replication, many therapeutic techniques have been administered, including antibiotics, antiviral medicines, and IL-6 inhibitors. However, the failure of all of these strategies led to the use of plasma transfusion and antibody cocktails to enhance an individual's immune system. Ethnopharmacological evidence supports anti-inflammatory properties of Bryonia, as an *in vitro* and *in vivo* study revealed that Bryonolic acid, an active component of the plant, upregulates the expression of heme-oxygenase-1 (HO-1). It also downregulates the expression of nitric oxide (NO), and inducible nitric oxide synthase (iNOS) in RAW264.7 macrophage cells. Thus, it is an excellent pathway to combat oxidative stress during inflammation (Ielciu et al. 2016, 2019). *Bryonia alba* was also found to suppress the development of B-16 melanoma cells *in vitro*, by activating heme oxygenase 1 (HO-1), and lowering nitric oxide (NO) levels (Gatbonton-Schwager et al. 2012). As because upregulation of HO-1 is linked to increased production of type-1 IFN, it may be considered as a potential therapeutic target. During the early stages of viral replication, the interplay between type-1 IFN and SARS-CoV-2, and their persistent attempt to repress one another, plays a significant role in immunomodulation (Barker et al. 2010; Singh et al. 2020; Konno et al. 2020; Ribero Sa et al. 2020). Hepcidin is a polypeptide hormone, and it is the only known cellular iron transporter, which shares a structural homology with SARS-CoV-2 spike protein (Hsani 2020). Altered immune response due to dysregulated iron metabolism, and post-capillary iron sequestration in the pulmonary endothelium among the patients, can be due to the former similarity between SARS-CoV-2 and hepcidin. As we know, overexpression of hepatic hepcidin is coupled with downregulation of ferroprotein, and therefore, HO-1 upregulation can suppress the production of hepatic hepcidin. This can limit the possibility of extracellular iron deposition (Puri et al. 2017). These findings not only support the use of HO-1 modulators, but they also give compelling evidence for the use of BA. In another study using virtual screening and molecular docking, Bryonolic acid and vitexin revealed a high and stable interaction with the receptor-binding sites of spike protein and ACE-2 receptor (Alagu Lakshmi et al. 2021). Here in this experiment, the upregulation of IFN genes with ethanolic extracts of Bryonia backs the possibility of implementation of HO-1 modulation in the SARS-CoV-2 spike protein RBD-induced hyper-inflammation in chick embryo. In the past, HO-1 inducers such as Celastrol and Andrographolide have been shown to increase type-1 IFN production and limit Hepatitis C virus replication (Blanco-Melo et al. 2020; Andrea et al. 2002). Upregulation of HO-1 has also been shown to have antiviral properties against Hepatitis B, Dengue, HIV, Ebola, and Zika virus (Tseng et al. 2017, 2016; Lee et al. 2014; Protzer et al. 2007; Gill et al. 2018; Hill-Batorski et al. 2013; Kalamouni et al. 2019). Hence, the finding of this study proves *Bryonia alba* can be considered as a potential alternative in the treatment of SARS-CoV-2.

## Conclusions

The above findings may be helpful in understanding scope and limitations regarding use of *Bryonia alba* in SARS-CoV-2 infections. This study also helps us to understand the curative and preventive effects of ethanolic extracts of *Bryonia alba.* The important findings of this study are significant upregulation of IFN-α, IFN-ß, TGF-ß by Delta SARS-CoV-2 spike protein RBD antigen, in concentration of 10 µg/mL. When Bryonia 6C was administered before administration of the antigen, due to upregulation of IL-6 it may not be beneficial; but due to opposite results when Bryonia 6C was administered after antigen, it may be beneficial to the biological systems. Histological studies also confirm almost normal condition of liver in this set. For future consideration, it should be noted that different phases of human trials are necessary before its final acceptance for treatment at large.

## Data Availability

All data and materials are allowed to be available for scientific studies.

## Abbreviations

SARS-CoV-2: Severe acute respiratory syndrome coronavirus type 2
RBD: Receptor-binding domain
CAM: Complementary and alternative medicine
IL-2: Interleukin 2
IL-6: Interleukin 6
IL-10: Interleukin 10
GM-CSF: Granulocyte–macrophage colony-stimulating factor
IP-10: Interferon-induced protein 10
MCP-1: Monocyte chemoattractant protein 1
MIP-1A: Macrophage inflammatory protein 1A
TNF-α: Tumour necrosis factor
BA: Bryonolic acid
